# Surgical treatment of parietal defects with “da Vinci” surgical robot

**Published:** 2012-06-18

**Authors:** D Vasilescu, S Paun

**Affiliations:** * Plastic Surgery Department, Clinical Emergency Hospital Bucharest, Romania; ** General Surgery Department, Clinical Emergency Hospital Bucharest, Romania

**Keywords:** robotic surgery, robotic surgery, incisional hernia

## Abstract

The robotic surgery has come through the development of telemedicine and minimally invasive surgery concepts, being developed in the military medicine by NASA during the years 1970-1980.

The purpose of this paper is to briefly present our experience in the new field of the robotic surgery, by analyzing the results obtained over a lot of 20 patients operated with the “da Vinci” robot within the last 5 years in the Clinical Emergency Hospital Bucharest for various abdominal defects

## Introduction

The presence of the abdominal wall defects especially that of the bulky ones, causes a series of disorders in both the abdomen and the thorax. They are: disorders of visceral statics; ventilation disorders; cardio-circulatory disorders; digestive disorders and disorders of the digestive transit; urinary disorders; disorders of some physiological acts involving the “abdominal press” [**1,2**]

### Classical surgical treatment

Over the time, the therapeutic concepts evolved and diversified, but, so far, a perfect and infallible procedure was not found. 

The failures obtained by using the procedures of simple suturing or of plasty (including myorrhaphy) led to the necessity of using some grafts (autografts or heterografts) conferring an additional mechanical resistance or the substitution of the parietal muscular aponeurotic structures. 

Two concepts were found:

o plasty of consolidation (in case of some qualitatively deficient muscular aponeurotic structures, which require the placement of grafts of consolidation); 

o the substitution graft in which the existence of a large defect or of a high eventrated volume does not allow the joining edge to edge of the margins of the wall defect.

At present, the surgical treatment of the abdominal wall defects registers a new phase, generated by the appearance of the minimally invasive surgery allowing the placement of the synthetic grafts made of polymers (preperitoneal) [**1,2**].

### Laparoscopic treatment of the abdominal wall defects

The success of the laparoscopic cholecystectomy extended the sphere of the laparoscopic approach to other abdominal surgery procedures, including the cure of the abdominal wall defects, especially of hernias. 

In comparison with the open surgery in which the adhesiolysis is achieved under manual control, laparoscopy has significant advantages:

- comparatively with the fixed and anterior image in laparatomy, the optics of laparoscopy offers enlarged images, both lateral and posterior, as well as the possibility to vary the visual field. 

- the epiploic appendages and the intestinal loops are distended by pneumoperitoneum between their mesenteric roots and the parietal adherences, easing the dissection very much [**1,2**].

By working carefully, gently and patiently the release of adherences of the whole abdominal wall is possible.

The limits of the laparoscopic parietoplasty: general anesthesia; local factors: the causes that determined the surgical procedure, the size; the operator’s experience; mechanical limits; biophysical limits [**2**].

### The surgical treatment of the parietal defects by using “Da Vinci” surgical robot

The robotic surgery appeared with the evolution of the concepts of telemedicine and minimally invasive surgery, being developed in the frame of the military medicine by NASA, in 1970-1980.

By this minimally invasive surgery, the image technology captures digital images of the surgical field, when the laparoscopic surgical instruments are introduced through the access ports at a certain distance from the operator. 

Da Vinci system also places the surgeon at a central console, but the 3 robotic arms are mounted on a mobile cart, with the central arm having 2 cameras and the lateral arm controlling the instruments.

The image is stereoscopic and the tips of the instruments perform a movement harmonizing with that of the surgeon’s hands, much more close to the mechanics of the open surgery than to the classic laparoscopic instruments [**3**].

## Patients and method

During January 2005 – January 2010, 20 patients were operated with the „Da Vinci” surgical robot for various abdominal wall defects, in the Clinical Emergency Hospital Bucharest.

In the statistical study the general data and the patients’ history: sex, age, comorbidities (obesity was defined as index of body mass), surgical history – operations that generated eventrations as well as preceding alloplastic parieto synthesis, were registered. 

The factors related to the surgical procedure, the operation time, the localization of the parietal defect, the surgical technique, prosthesis positioning (supra-aponeurotic, properitoneal, intraperitoneal), the type of prosthesis used, the features of the surgical technique or associated surgical techniques, particular special situations (the special situations were represented by patients with histories of several times relapsed eventrations and alloplasties, that needed the removal of the old prosthesis together with the compromised tissues, as well as by patients with varying degrees of obesity), were studied.

The postoperative care was common both for the patients with operated eventrations and the patients with associated abdominoplasty and consisted in: 

- anticoagulants and broad-spectrum antibiotics

- nasogastric probe 

- early mobilization.

The patients were monitored throughout the hospitalization period, with a recording of the early and late postoperative complications, as well as of the therapeutic methods that were used.

## Results 

As far as the sex distribution is concerned, we have found the predominance of the feminine sex. In our study, women accounted for 59,69% compared with 40,30% men. 

Age. The mean age of the patients who took part in the study was of 58 years, with extremes between 19 and 81 years.

47% showed monosaccular eventrations (the diastasis of rectus abdominis muscles being presented constantly in this manner) and 33,5% presented multiple sac eventrations. 

Etiopathogenicity of the postoperative eventrations

In 53% of the total number of eventrations, including the relapses, the pathological process causing the occurrence of eventration was wound suppuration. 

Obesity – degree II-III was also an important factor: 49,4% 

Coughing chronic respiratory diseases were found in 17% of cases and cardio-vascular diseases in 45,2%. The non-observance of the effort regime postoperatively was identified anamnestically in 14,6% of cases. 

Preoperative preparation. Paraclinical investigations:

- biological samples; 

- radiological exploration; 

- urography and cystography; 

- abdominal ultrasound exam; 

- computer tomography; 

- exploration of ventilatory lung function.

**General preparation**

It aims to the balancing and improvement of the patient’s metabolic condition: nutritional balance, associated diseases. The most common problems encountered in patients with eventrations are obesity, chronic lung diseases, cardiovascular diseases, obstructive minor ailments.

**The local preparation** of the patients with postoperative eventration had as a goal the sanitation of the infection foci (parietal granulomas).

**Complications**

**Table 1:** Life threatening postoperative complications

Complication	Nb
Thrombo-embolic accidents	0
Bowel obstruction	0
Evisceration	0
Peritonitis	0

**Table 2:** Immediate complications

Complication	Nb
Hemorrhage	0
Hematoma	0
Restrictive respiratory failure	0
Thrombo-embolic accidents	0
Ischemia and flap necrosis	0
Wound suppuration	0

**Table 3:** Late postoperative complications

Complication	Nb
Bowel obstruction	0
Evisceration	0
Digestive fistula	0
Peritonitis	0
Relapse	0
Seroma	0

Mean duration of hospitalization: 1 day 

Relapse rate 1 year postoperatively: 0 cases

Clinical Case: Patient M.N., male, 4 years old, no comorbidities: Umbilical hernia repair with da Vinci robot

Intraoperative images

**Fig. 1:**
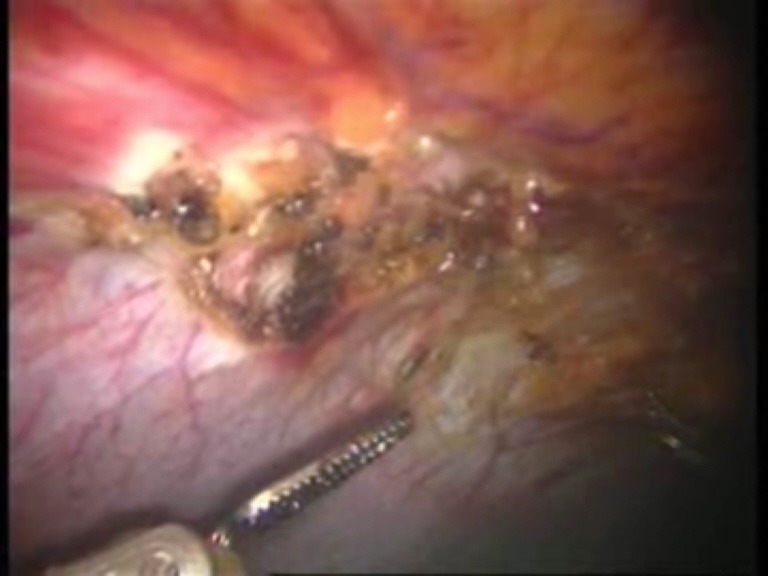
Umbilical defect

**Fig. 2:**
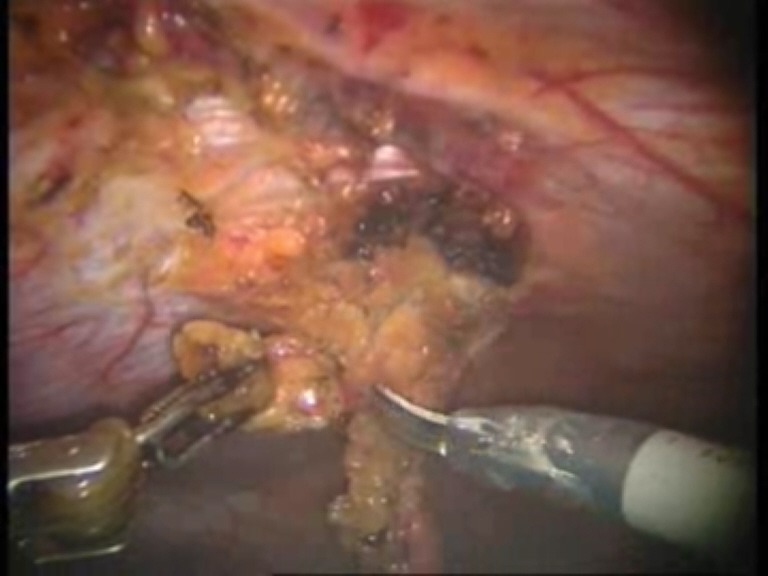
Viscerolysis of the parietal defect

**Fig. 3:**
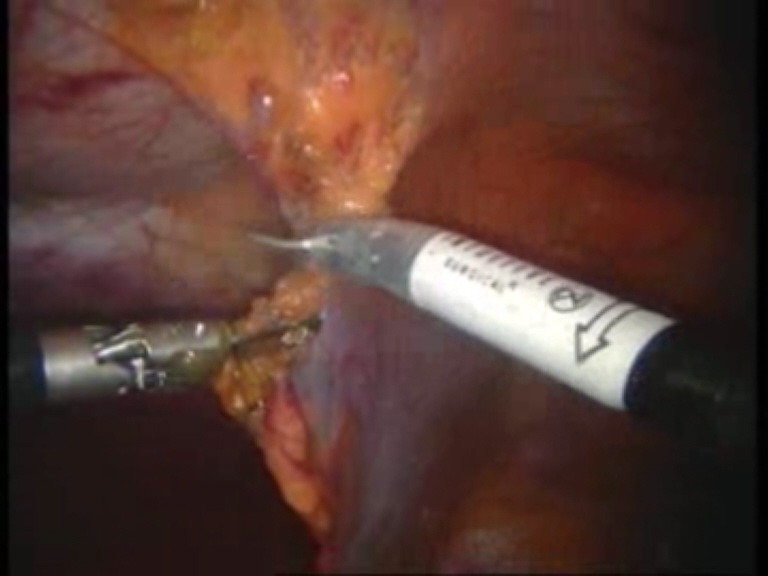
Detachment of the falciform ligament

**Fig. 4:**
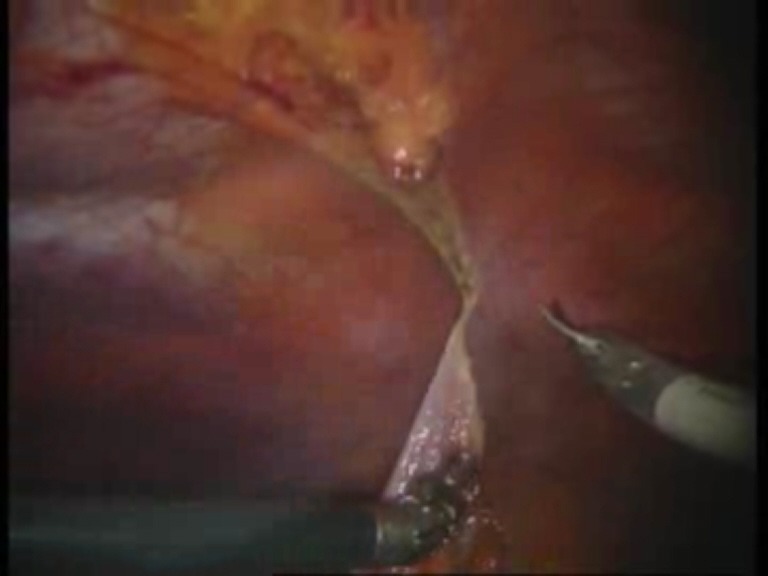
Final detachment of the falciform ligament

**Fig. 5:**
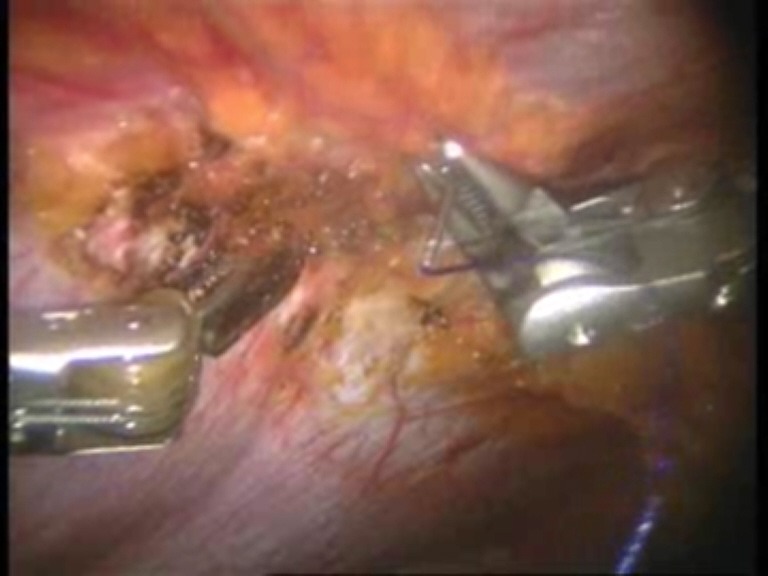
First suture thread passed through the defect margin

**Fig. 6:**
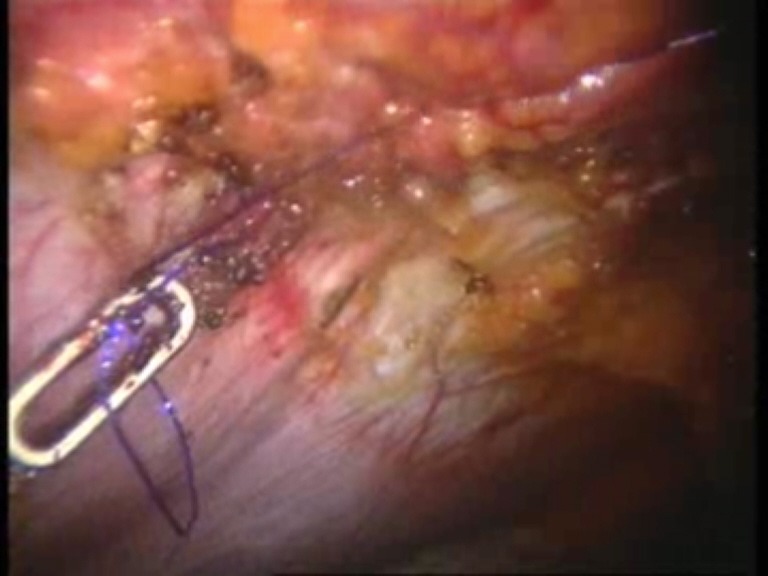
First suture thread knotted

**Fig. 7:**
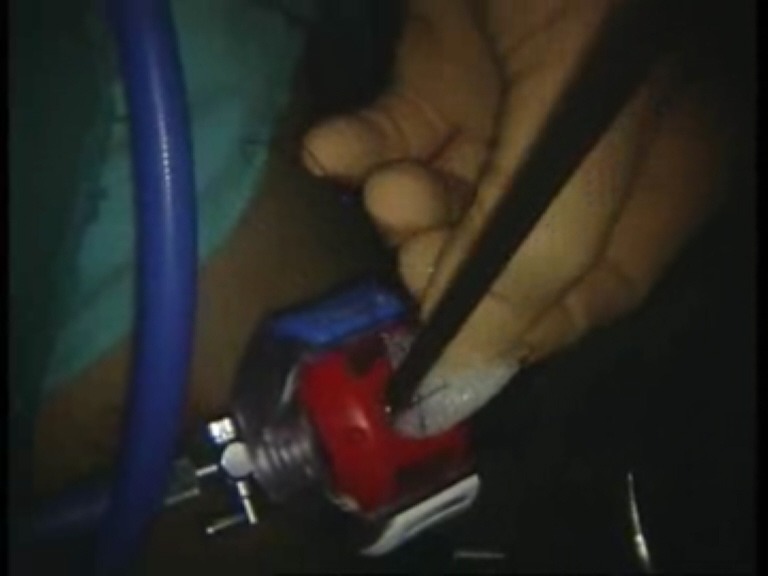
Introduction of the mesh on the trocar

**Fig. 8:**
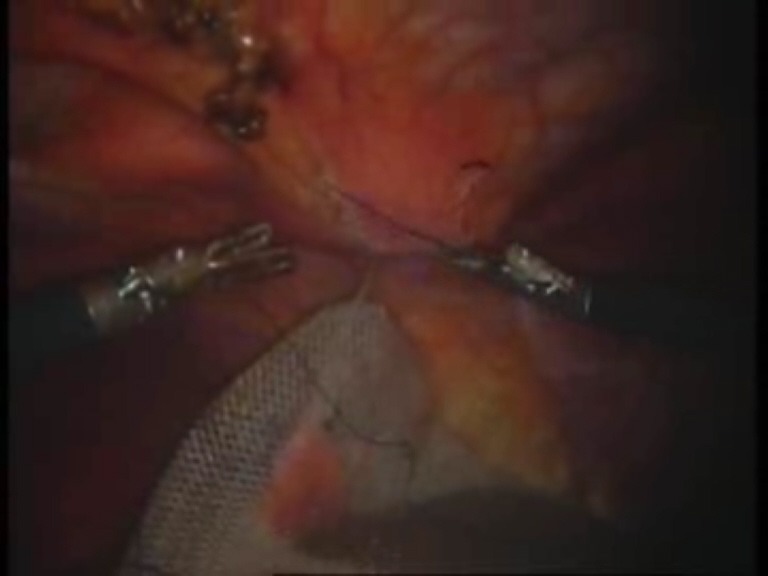
The first suture thread passed transparietally

**Fig. 9:**
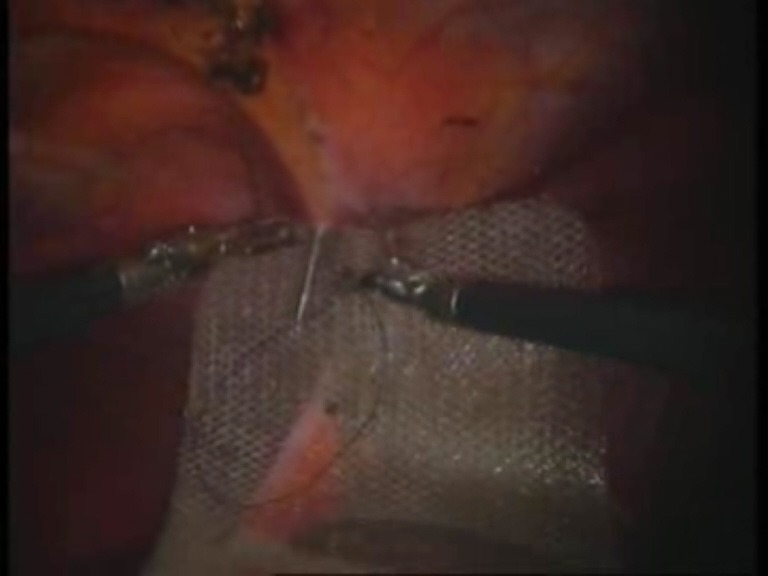
The first suture thread (the second arm) passed

**Fig. 10:**
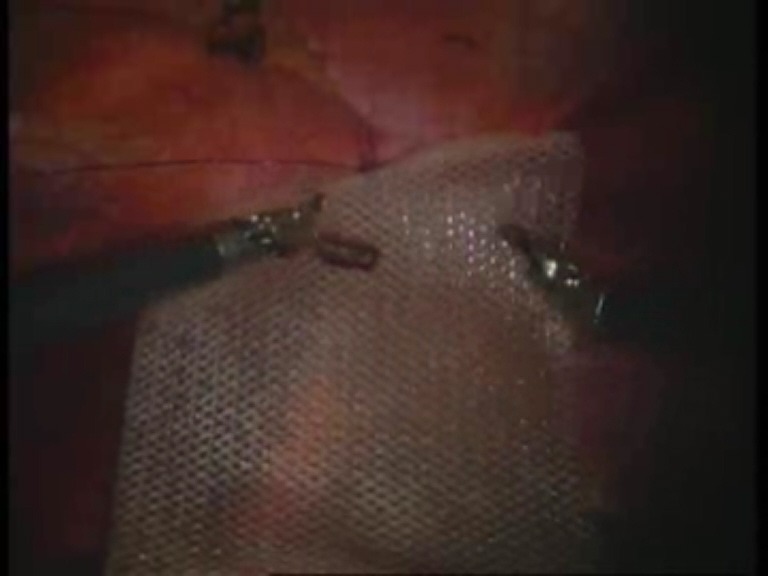
Mesh fixed with the first suture thread

**Fig. 11:**
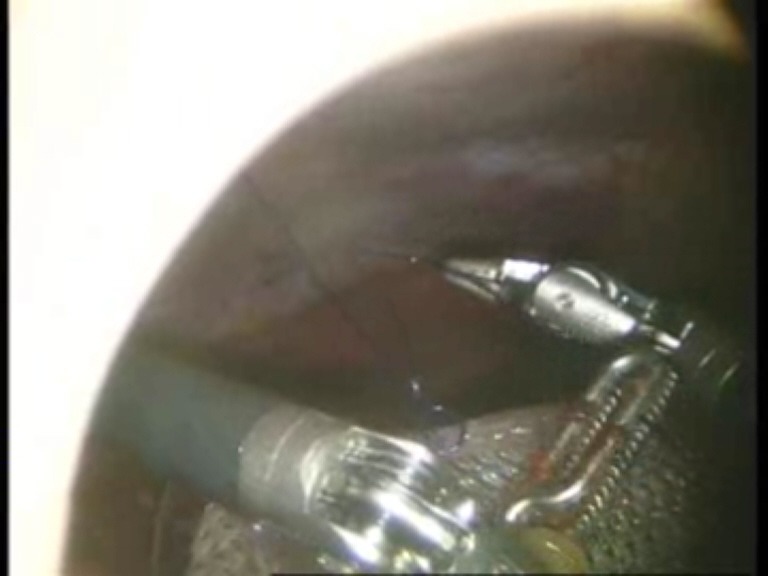
The second suture thread for mesh fixation passed transparietally

**Fig. 12:**
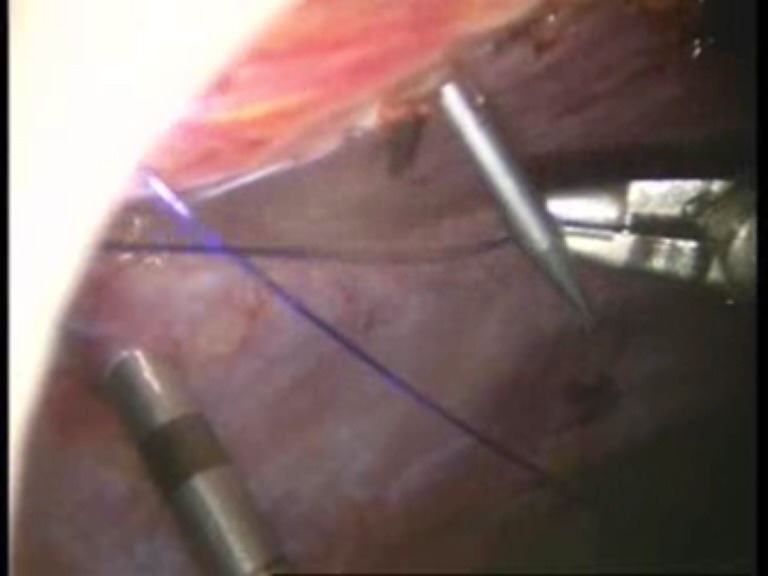
The second suture thread (the second arm) passed in a transperitoneal manner with Reverdin’s needle

**Fig. 13:**
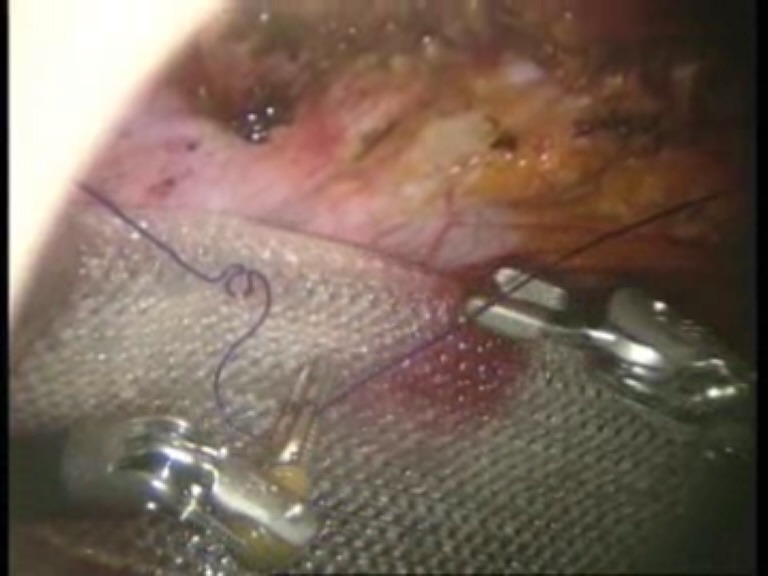
Mesh fixed over the parietal defect with the second suture thread – mesh arrangement

## Discussions

The term “robot” was introduced by the Czech playwright Karel Capek in 1932, through a play in which the robots took over the human race. There was a true explosion of “SF industry” in the years that followed, in which the robots were presented from friendly companions to evil entities. 

In reality, robots have revolutionized many branches of the industry, starting with the cars’ industry and ending with the pharmaceutical one. 

Unlike the autonomous robots in SF films, the industrial robots are controlled by computer programs, to fulfill accurate tasks. Therefore, they entered the public consciousness either as a SF curiosity or as mechanical machines driven by digital systems, without human intervention.

The surgical robots have nothing in common with the industrial ones, being actually an “extension” of the surgeons’ mind and hands or definitely said, surgical instruments handled remotely through a console by the physician. They are neither autonomous nor driven by computer programs. 

According to the minimally invasive surgery, the image technology captures digital images of the surgical field, when the laparoscopic surgical instruments are inserted through access ports at a certain distance from the operator. Having the potential to overcome the limitations of conventional videoendoscopic surgery:

1. 3-D visualization—Moreover, the robotic camera can be controlled by the surgeon, and held in a steady position without fatigue or delay in movement 

2. Endowrists—The robotic instruments respond as though the surgeon's fingertips are at the end of the instruments and are directly manipulating the specific instrument at the point of impact with the tissue.

3. Scaling of instrument movement and tremor filtration—Robotic systems use software that electronically remove tremor and allow for large, coarse motions of the surgeon's hand to be translated into fine movements with the help of the instruments [**1**].

The cumulative effect of the robotic systems is precision and dexterity (that surpasses conventional videoendoscopic systems and even the human hand). In general, robotic surgical systems are extremely fine [**4-6**].

Initially, the application of laparoscopic surgery was enthusiastically received, having the following benefits: 

- minimal incisions 

- reduced postoperative morbidity 

- diminution of the hospitalization time 

The disadvantages are: 

- length of instruments

- stereoscopic loss of the perception of depth sensation 

- loss of tactile sensation 

- increase of tremor [**3-7**]

In addition, the manipulation of laparoscopic instruments requires a suitable training, as their tips should be directed opposite to the actual hand movement. 

Briefly said, the minimally invasive surgery requires a very good coordination of the surgeon’s sensory, spatial and psychomotor qualities. 

Some of these constraints of the minimally invasive surgery can be solved through robotic surgery in which computerized manipulators handle the laparoscopic instruments in the surgical field. Therefore, it was suggested that the laparoscopic surgery techniques have played a transitional role for the Robotic Surgery, with the following advantages: 

- diminution of tremor

- three dimensional view 

- high precision 

- limitation of errors 

- diminution of the fatigue due to increased comfort [**3**].

The robotic system offers performance accompanied by stability and accuracy, with a perfect duplication of the surgeon’s hand.

The development of the robotic techniques for general surgery was possible through the cooperation of engineers, IT specialists and doctors. The beginnings of their use are in 1988, when the Probot unit was designed for transurethral surgery. In 1991, IBM specialists created the Robodoc unit was for hip prostheses, as well as for prostate transurethral resection.

The first active robotic device for the placement of an endoscopic camera, called Automatic Endoscopic System for Optical Positioning (AESOP, Computer Motion, Goleta, CA) was approved by FDA in 1993. NASA made its development for space programs and it was originally controlled by a foot pedal, AESOP evolving in 1996 to a voice command [**3**]. 

The major benefit of AESOP and of its laparoscopic port (Alpha port) is represented by the stability of the captured image, which allows the integration of a transmission of 15 images per second to an audience situated away from the surgical field [**3**]. 

The robotic systems for general surgery are an adaptation of their original development for cardiac surgery. ZEUS system was designed for minimally invasive surgery procedures as endoscopic coronary bypass (E-CABG) and mitral valve surgery and the da Vinci® system was created for coronary artery bypass. 

The functional aspects of the robotic surgery can be used to classify the robotic systems in pre-programmed or telemanipulated systems [**3**].

**Fig. 14:**
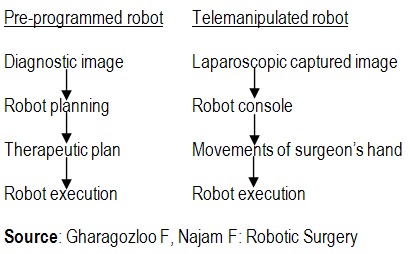
Source: Gharagozloo F, Najam F: Robotic Surgery

The development of the possibilities to use real-time images during minimally invasive surgery procedures led to the emergence of either Endo Assist type robots, which position the camera by using infrared sensors to detect the surgeon’s hands movements or AESOP type robots using voice control.

The applications in general surgery have gradually expanded, reaching a very broad spectrum that includes cholecystectomy, Nissen fundoplication, Heller myotomy, gastric bypass, adrenalectomy (for which it is the gold standard), resection of bowel loops, esophagectomy [**2**]. 

There are two types of surgical robots approved by the FDA, for the surgeons’ use: ZEUS (Computer Motion) and da Vinci Surgical System. 

Both systems have multiple robotic arms, controlled by the surgeon from a computerized console, remotely located, with video-assisted visualization. 

The ZEUS robotic system places the surgeon at a central console that communicates with three robotic arms. Two of them handle the surgical instruments, while the central one functions as a “laparoscopic camera holder” [**8**]. 

The surgeon controls this system by voice.

Da Vinci system also places the surgeon at a central console, but the three robotic arms are mounted on a mobile cart, the central arm having two cameras and the lateral arms controlling the instruments. 

The image is stereoscopic and the tips of the instruments perform a movement harmonized with the surgeon’s hand, much closer to the mechanic of the open surgery than the classical laparoscopic instruments. 

Comparing the two systems, Sunny et al. noted that both units are similar in reducing tremor and the surgeon’s fatigue, but da Vinci system is more intuitive as far as tasks performance is concerned [**7**].

Dakin and Gagner have compared the two systems with standard instruments for laparoscopy, which were used for basic actions such as suturing. 

The smoothness of the suture and its precision were clearly superior for the robotic systems [**3,7**].

## Conclusions

Although very tempting and spectacular, the cure of parietal defects with surgical robots is far from being imposed as a large-scale therapeutic method, firstly because of the costs and of the prohibitive infrastructure at present, but also because they have not been demonstrated results superior to those obtained by laparoscopy.

The integration of robotic surgery into operating room practice has a short history, and currently less than 300 systems have been installed worldwide [**3,9**]. Robots with interaction between human and machine will become more natural, as the technology evolves, in translating the surgeon's motions to end-effectors. Robots can access small spaces, scale the motions from nano to macro, interpret preoperational images, capture incredible amounts of information relative to the surgical event, and integrate distant students or consultants with seamless telecommunications. Technology can be evolutionary at a variable rate as in biology. For example, the broad application of laparoscopy in gynecology preceded the recognition in general surgery for cholecystectomy after 20 years [**10**]. The revolutionary change or disruptive technology may lead to immediate change in the practice in coronary artery bypass, [**11**] or the technology may lie fallow for decades before the applications are found. Robotic surgery is feasible, established, and has a strong advocacy from a growing user community. Surgical robots are, however, very expensive in a time of resource constraint in medicine. One may assume that the future of robotics in general surgery will be characterized by practicality and response to clinical need. Finally, surgical robots will have a broad application and financial success when the public imagination is fully engaged and the public demands this elegant and promising technology [**3**].
